# Editorial: Coronavirus Disease (COVID-19): The Mental Health, Resilience, and Communication Resources for the Short- and Long-term Challenges Faced by Healthcare Workers

**DOI:** 10.3389/fpsyg.2022.904328

**Published:** 2022-04-18

**Authors:** Andrew E. P. Mitchell, Federica Galli, Chris Keyworth, Elena Vegni, Eduardo Salas

**Affiliations:** ^1^Faculty of Health and Social Care, University of Chester, Chester, United Kingdom; ^2^Department of Dynamic and Clinical Psychology, Sapienza University of Rome, Rome, Italy; ^3^School of Psychology, University of Leeds, Leeds, United Kingdom; ^4^Department of Health Sciences, University of Milan, Milan, Italy; ^5^Department of Psychology, Rice University, Houston, TX, United States

**Keywords:** coronavirus disease (COVID-19), mental health, communication, resilience, health care workers (HCWs)

During the early phases of the COVID-19 pandemic, the world initially focused on measures to suppress COVID-19 transmission and protect their populations by developing vaccines and drug treatments for the most vulnerable and a host of social actions, including implementing social distancing, working from home, travel restrictions, lockdowns, and face coverings. Nearly 2 years after the initial outbreak, at the time of writing this editorial, and through research conducted as part of this Research Topic, it is clear that the mental health impacts of COVID-19 on healthcare workers (HCW) are significant. There is an urgent need to understand and address these impacts (Greenberg et al., 2020). This is particularly true given the World Health Organisation has outlined a series of mental health and psychosocial considerations aimed explicitly at HCWs (World Health Organisation, 2020). The present Research Topic on Coronavirus Disease (COVID-19) and HCWs has added to the scientific knowledge in several main areas, including barriers and enablers to healthcare delivery, understanding HCWs' mental health and well-being, resilience, coordination and communication within the workforce, and specific interventions to promote mental health and well-being. The Research Topic yielded 42 articles with contributions from 240 authors. The articles within this Research Topic were published between the third quarter of 2020 and 2021. The majority of studies were conducted in Europe (*n* = 26), with most conducted in Italy (*n* = 13), the United Kingdom (*n* = 3), and Spain (*n* = 3). There was also one study from each of Norway, Denmark, Romania, Turkey, Portugal, Austria and Switzerland. Asia included China (*n* = 6), India (*n* = 3), one study in Pakistan and Vietnam, one study conducted in Brazil, and four studies in the United States of America.

The heterogeneity of the studies in terms of location and populations further contributes to the Research Topic. The study designs can be dichotomised, with the majority of studies (*n* = 29) being cross-sectional. Most were questionnaire studies in which a population is surveyed at one point in time to describe characteristics. Other studies (*n* = 5) were broadly qualitative and used interviews or focus groups. There were systematic reviews (*n* = 5), mostly narrative reviews and one example involving meta-analysis (Dong et al.). There was one randomised control trial reported within the Research Topic (Procaccia et al.). Finally, there was a mixed-method (Putrino et al.) and an opinion/commentary paper (Chapman et al.). There was a range of analysis techniques in the qualitative papers. The most frequent method was to conduct interviews, with most using thematic and less frequently involving content analysis. Data analysis within the quantitative papers used descriptive and dispersion analysis, analysis of variance, regression analysis and factor analysis to report the results. The submissions assessed various mental health outcomes, including anxiety and mood disorders, post-traumatic stress disorder, obsessive-compulsive disorder, and sleep disturbance.

The different studies collected in the Research Topic may be described according to four lines of research. Firstly, a part of the studies addressed the enablers and barriers in healthcare delivery, both person-specific variables and resources to deliver healthcare. Moreno-Jiménez et al. utilised the Job Demands-Resources model (JD-R; Bakker and Demerouti, 2017) and reported that high job demands by HCWs during the COVID-19 pandemic were related to a lack of appropriate resources, such as protective equipment in the healthcare environment. The limited supply or lack of Personal Protective Equipment (PPE) was related to adverse outcomes, including increased workload and fear of contagion. The authors suggested that increased resources such as PPE could reduce fear of contagion and emotional consequences. It has been found that COVID-19 can affect team performance at four stressor levels: individual, team, organization, and work-life (Tannenbaum et al., 2021). Working in healthcare settings during a pandemic has the potential risk to cause high levels of stress because of exposure to a range of potentially stressful situations.

Some specific stressors for HCWs have included the interpersonal aspects of practise, clinical environment, keeping up to date with current knowledge and dealing with patient concerns (Mitchell, 2020a). A study by Del Piccolo et al. focused on individual, interpersonal and organisational resources to reduce stress. The authors suggested that the essential aspects are the promotion of acceptance of negative emotions and resilience to stressors at the individual level. At the interpersonal level, peer support and daily sharing of experiences helped. At the organisational level, the findings suggested that access to COVID-19-specific resources, such as PPE, enabled Italian obstetrics staff to undertake their work safely whilst reducing distress. Healthcare workers' health and welfare are important resources and potential barriers. Individual well-being was described in two papers (Raza et al.; Testoni et al.) by investigating the lived experiences of health workers during the COVID-19 pandemic. Both studies utilised qualitative interviews in different countries and found that frontline workers experienced the highest personal distress when confronted with COVID-19.

The second aspect of the Research Topic focused on articles investigating HCWs' mental health and well-being during the first 18 months of the COVID-19 pandemic. Galli et al. (2020) reported the likely risk of developing a psychiatric disorder for healthcare workers during the pandemic. An article by Chatterjee et al. found that 79.3% of the HCWs had moderate to severe levels of perceived stress, and 47.9% had insomnia during the early phase of the pandemic in India. Huo et al. studied the determinates of burnout of HCWs during the COVID-19 pandemic in China. The authors indicated that 36.5% of workers experienced burnout. The findings highlighted personal and work-related factors were associated with burnout, such as being less experienced HCW and younger. Another study in the United States by Pearman et al. found that healthcare workers were at an increased risk of experiencing mental health issues such as depression and anxiety compared to a matched general population sample during the pandemic. Furthermore, the authors indicated that HCWs, on average, had a symptom profile to reach a clinical diagnosis of depression. Pfefferbaum and North (2020) reported that HCWs are at risk due to job-specific attributes, i.e., exposure to disease and concerns about transmitting the infection. Early and mid-term consequences on HCWs' physical, behavioural, and mental health were focused on by Khanji et al. by developing a study protocol (CoPE-HCP) to compare HCWs and the general public. The authors hoped to improve the delivery and design of support systems for HCWs and the public.

A third aspect relates to articles addressing resilience and communication themes. This aspect attracted research investigating the adherence and understanding of clinical guidelines and the impact of the pandemic on levels of emotional distress and resilience of HCWs. Outside of this Research Topic, Keyworth et al. (2021) investigated adherence to Government guidelines in the general population and reported that the psychosocial effects could undermine long-term adherence. Riguzzi and Gashi examined the psychosocial lessons learnt during the first wave of COVID-19 and adherence to guidelines in HCWs in Switzerland. The authors found a high level of emotional distress, with 70% of the HCWs reporting emotional distress in the first pandemic wave. Fifty-two percent of HCWs felt worried about passing the virus on to their family or friends. In contrast, 18% of HCWs felt worried about the same happening to themselves. The findings also suggest an overestimation of the effectiveness of standard hygiene procedures, with 36% falsely believing standard hygiene measures would keep themselves and others safe. Lenzo et al. focused on the relationship between emotion regulation and its effect on depression and anxiety. The authors found that perceiving stressor context cues was inversely associated with depression and anxiety. This finding suggests the possibility of using psychological theories to support psychological interventions to help mitigate the psychological consequence of depression and anxiety. The authors did not name a specific intervention but named a broad range of third-wave cognitive and behavioural techniques such as mindfulness-based interventions to decrease compassion fatigue and resilience amongst HCWs (Zhang et al.). The relationships between mindfulness and resilience have been studied by Mitchell (2020b), finding that acceptance and attention within mindfulness was important for HCWs' resilience.

The last main grouping of articles focused on specific interventions to promote mental health. Callus et al. completed a rapid review to identify the most effective stress reduction techniques for healthcare workers managing infected patients with coronavirus (SARS, MERS, and COVID-19). The authors identified several studies focusing on interventions to support HCWs. Still, most did not test user satisfaction or conduct a follow-up, which suggests a need for further research into stress reduction interventions to safeguard HCWs' mental health. This area of research is needed to protect staff from fatigue and burnout during high levels of acknowledged exposure to stressors during the pandemic (Leo et al., 2021). Callus et al. reported on a digital package in which user satisfaction was measured (Blake et al., 2020). The evaluation of the online support package indicated a high user satisfaction for content, usability and utility amongst HCWs in the United Kingdom. In another study, Putrino et al. showed that after a single 15-min experience in a multisensory experience recharge room, healthcare workers showed a 59.6% reduction in self-reported stress levels and rated the experience positively at 99.3%.

Studies have also looked at service-level implementation by teams in response to COVID-19. A study by Cao di San Marco et al. (2020) reported implementing a clinical psychology service and detailed two types of psychological support, decompression rooms and small-group sessions, as beneficial. A similar service-level provision was reported by You et al. focussed on hotline counselling service, which was set up following the initial COVID-19 outbreak to provide HCWs with psychological support. The authors devised a psychological hotline scale to assess skills and reported a good level of reliability and validity. The scale was designed to screen and evaluate the competencies of counsellors providing hotline support. Aristizabal et al. reported on heart rate variability biofeedback to support HCWs at times of stress and anxiety. The authors highlighted that diaphragmatic breathing exercises could positively reduce stress and anxiety. Procaccia et al. investigated the benefits of expressive writing compared to neutral writing on HCWs' psychological adjustment during the COVID-19 pandemic after three writing sessions. The findings suggest a positive benefit in psychological adjustment to several psychological outcomes.

## Conclusion

In summary, this Research Topic has gathered articles from around the world and focused on HCWs and the best evidence to support their mental health and well-being during the pandemic. The studies report from the meso-level of organisations to the micro-level of individual behaviour and cognitions. These articles have contributed to the understanding of the needs of the HCWs to deliver health in the most effective and safe ways for the patients whilst protecting themselves as an invaluable resource.

This Research Topic has published studies addressing a range of topics relevant to understanding mental health, resilience, coordination and communication within the workforce, and specific interventions to promote mental health for HCWs during the COVID-19 pandemic. Coronavirus is likely to be a challenge for the foreseeable future regarding understanding its sequelae for the HCWs themselves. Future consideration of well-being and mental health is needed amongst frontline workers. There is a need to understand how to prevent distress and provide interventions to support healthcare workers during such periods. This Research Topic is a valuable source for future work in the area. Hopefully, this Research Topic will motivate more research on this important worldwide topic.

## Author Contributions

AM wrote the initial draft. FG, CK, EV, and ES critically reviewed and provided valuable feedback on the final version of the manuscript. All authors approved the submitted version.

## Conflict of Interest

The authors declare that the research was conducted in the absence of any commercial or financial relationships that could be construed as a potential conflict of interest.

## Publisher's Note

All claims expressed in this article are solely those of the authors and do not necessarily represent those of their affiliated organizations, or those of the publisher, the editors and the reviewers. Any product that may be evaluated in this article, or claim that may be made by its manufacturer, is not guaranteed or endorsed by the publisher.
